# An absorbance method for analysis of enzymatic degradation kinetics of poly(ethylene terephthalate) films

**DOI:** 10.1038/s41598-020-79031-5

**Published:** 2021-01-13

**Authors:** En Ze Linda Zhong-Johnson, Christopher A. Voigt, Anthony J. Sinskey

**Affiliations:** 1 Department of Biological Engineering, Massachusetts Institute of Technology, 500 Technology Square, NE47-140, Cambridge, MA USA; 2 Present Address: Department of Biology, Massachusetts Institute of Technology, 77 Massachusetts Avenue, Building 68-370A, Cambridge, MA USA

**Keywords:** Biochemistry, Biological techniques, Biotechnology, Chemical biology, Microbiology

## Abstract

Increased interest in poly(ethylene terephthalate) (PET)-degrading enzymes (PETases) have generated efforts to find mutants with improved catalytic activity and thermostability. Here, we present a simple and fast method to determine relative enzyme kinetics through bulk absorbance measurements of released products over time. A thermostable variant of PETase from *Ideonella sakaiensis* was engineered (R280A S121E D186H N233C S282C) with a denaturation temperature of 69.4 ± 0.3 °C. This was used to assess the method’s ability to determine relative enzyme kinetics across variants and reveal structure–function relationships. Measurements at 24 and 72 h at 400 nM of enzyme suggest that the mutations improved catalytic rates 5- to 7-fold. On the contrary, kinetic analyses of the thermostable variant and wild-type reveal different reaction trajectories despite similar maximum catalytic rates, resulting in higher product accumulation from the thermostable variant over time. The results of the assay support the necessity for kinetic measurements to determine relationships between sequence and function for *Is*PETase and other PET hydrolases.

## Introduction

Plastic pollution has received increasing media attention and public concern as both its environmental and potential health hazards are being uncovered^1,2^. Without immediate interventions in plastic usage and industrial-scale changes to the intended life cycle of plastics, plastic consumption is projected to grow 300% between 2014 and 2050^3^. To address this exponentially growing crisis, researchers have begun to turn attention towards enzyme-based strategies for recycling and bioremediation^4–9^. Careful kinetic studies must be performed to identify accurate structure–function relationships for plastic-hydrolase engineering, but analyses of the initial phases of the reactions are not commonly reported, sometimes leading to misinterpretation of effects of mutations.

One of the most common plastic resins is poly(ethylene terephthalate) (PET), recognized by the “1” recycling label found on manufactured plastics. It is the primary material used to make plastic bottles and polyester textiles^10^. In 2016, research groups in Japan discovered a bacterium, *Ideonella sakaiensis*, that was able to utilize PET as a carbon source^11^. The organism produces an enzyme, *Is*PETase, that degrades PET polymers into terephthalic acid (TPA), mono(2-hydroxyethyl) terephthalate (MHET), and ethylene glycol, with minor amounts of bis(2-hydroxyethyl) terephthalate (BHET). These components can be recycled into new PET material or utilized as carbon sources by microorganisms. Research has since followed to characterize *Is*PETase with improvements to its activity and thermostability^7,12–15^.

*Is*PETase has been crystallized and shown to be a cutinase belonging to the serine hydrolase superfamily^12,13,16^. The working model for enzymatic attack of PET is through adsorption to the PET surface and binding of a segment of the polymer in the catalytic pocket of the enzyme^12,17^. Thus, enzyme engineering efforts for PET hydrolases have focused on improving thermostability, activity, and adsorption to PET^4,5,7,9,12,18–21^. For example, Oda et al*.* observed that the addition of calcium ions stabilized the PETase homolog Cut190*, and co-crystallization with calcium ions revealed three calcium binding sites. One of the calcium binding sites was replaced with a disulfide bond that resulted in a 23.1 °C stabilization of Cut190*^5^. A similar increase in stability was observed by Then and co-workers when the calcium binding site was replaced with a disulfide bond in TfCut2 from *Thermobifida fusca*^22^. However, increases in product formation from engineered enzymes have often been observed using endpoint quantification of supernatant product concentrations, and structure–function relationships have mostly been inferred based on these measurements.

The most established method in the field to detect PET degradation products is based on high performance liquid chromatography (HPLC)^5,7,9,11,12,13,22^. This method is accurate and sensitive to different products released during PET degradation and is extremely important for degradation analyses, but it is limited in terms of speed, throughput, and ease of setup to measure enzyme kinetics “continuously” from the same enzyme reaction. Complications arise when multiple parallel and continuous enzymatic reactions are to be sampled with HPLC, as sufficient volumes must be taken from the reaction supernatant for HPLC measurements. This decrease in volume of the enzyme reaction may become significant when > 1% of supernatant is collected for each time point. All these considerations make optimizing the HPLC method for “continuous” kinetics monitoring tedious and potentially inaccurate. Thus, endpoint measurements have been performed using HPLC, such that kinetics during the time course of the reaction remain undetermined. Nevertheless, kinetic studies have been shown to be critical in understanding enzyme function in heterogeneous-phase reactions, such as cellulases, which are known to exhibit decreasing reaction rates over time^23–26^. The decreasing rates are hypothesized to be related to enzyme interactions with the solid substrates and therefore fundamental to catalysis^26,27^.

A variety of continuous-mode methods for kinetic analysis of PET degradation have been developed for either surrogate PET substrates or PET nanoparticles, as well as fluorescence detection methods based on modified TPA produced from enzyme reactions with PET substrates^28–30^. These are limited in use for larger-sized PET films (a popular substrate in the field and relevant pollutant) or for PETase variants that generate considerable quantities of MHET and other degradation products. Another method for measuring reaction kinetics on PET films uses sodium hydroxide (NaOH) titration with a pH–stat as an indicator of MHET and TPA evolution, which are acidic, while neutral products such as BHET are not detected^31^.

In this report, we present a simple, fast, small volume, and resource-efficient method that provides insight into enzyme kinetics over time based on the absorbance of PET degradation products. Furthermore, a comparison was made between a thermostable mutant of *Is*PETase and wild-type *Is*PETase as a case study to demonstrate the method’s ability to provide insight into changes in enzymatic behavior due to thermostable mutations. The results of the study demonstrate that bulk absorbance can be used to determine relative kinetic profiles of PET hydrolases, and thermostable mutants can produce more product over time without improvements in catalytic rate.

## Results

### Generation of a thermostable *Is*PETase mutant for kinetic case study

**Figure 1:**
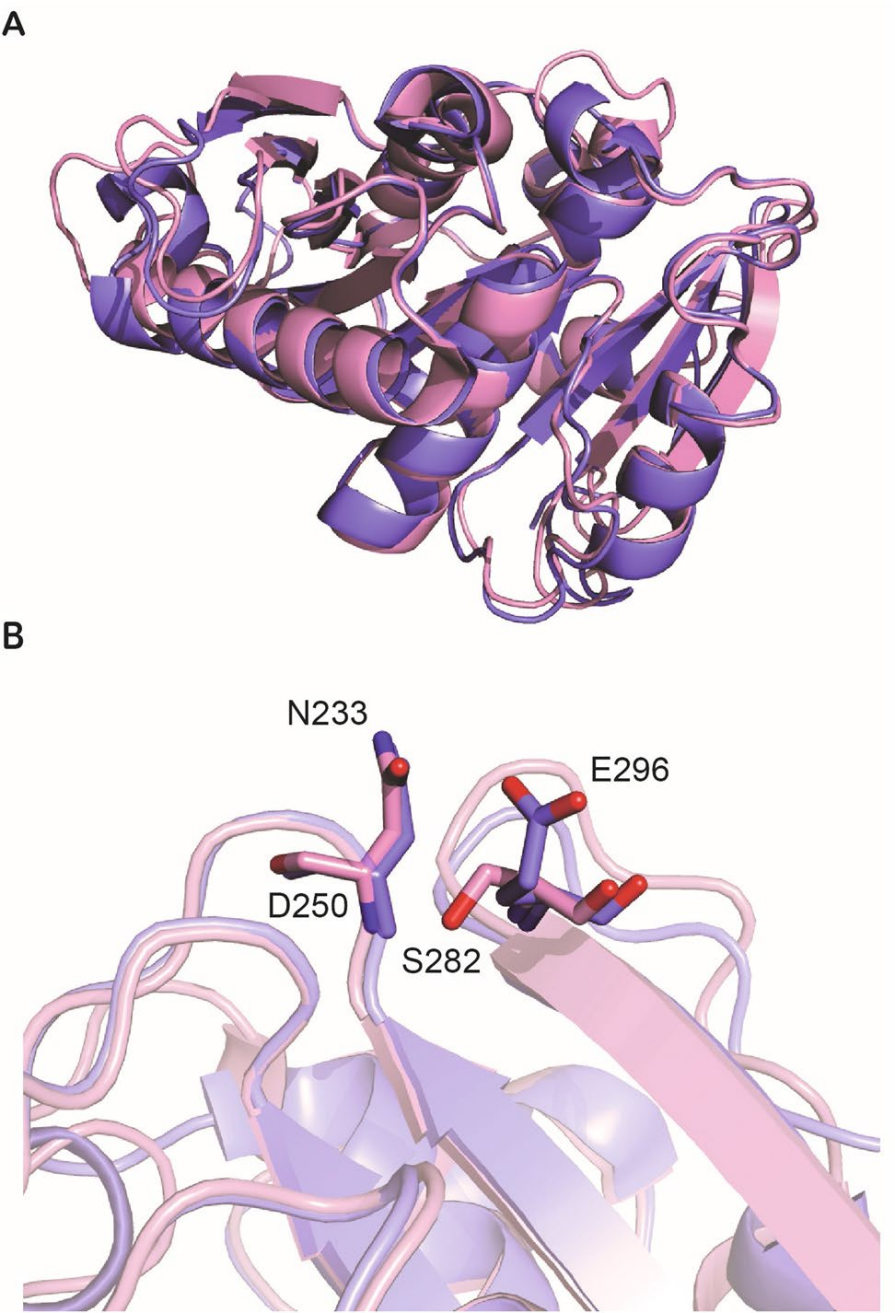
Homology alignment between *Is*PETase and Cut190*. (**A**) PyMOL structural alignment of Cut190* with *Is*PETase. MacPyMOL (ver 1.8.6.2) was used to perform the structural alignment^32^. *Is*PETase is pink (PDB: 6EQE^13^); Cut190* is blue (PDB: 5ZNO^5^). (**B**) Residues mutated to generate disulfide bridge in Cut190* (E296 and D250) align with S282 and N233 in *Is*PETase.

The structures of *Is*PETase and the PETase homolog Cut190* were aligned using MacPyMOL (version 1.8.6.2), revealing very similar folds with a mean root mean square deviation of 0.576 Å for an alignment of 190 backbone Cα atoms (Fig. 1A)^5,13,32^. The thermostability of Cut190* was improved 23.1 °C by mutating D250 and E296 to cysteines to generate a disulfide bridge^5^. Due to the similarity in structure, *Is*PETase residues N233 and S282 were mutated to cysteines, forming a disulfide bond between β-strands 8 and 9 (Fig. 1B). Differential scanning fluorimetry measurements showed that these mutations improved thermostability by 14.1 ± 1.3 °C, and when combined with a group of mutations known in literature (R280A, S121E, and D186H, *T*_m_ increase of 8.8 °C)^7,12^, a final *Is*PETase mutant with a denaturation temperature of 69.4 ± 0.3 °C was generated. The disulfide bond in *Is*PETase is novel in this study and results in one of the highest denaturation temperatures reported for *Is*PETase (Table 1, Supplementary Fig. 1). *Is*PETase R280A S121E D186H N233C S282C will be herein referred to as ThermoStable-PETase, or TS-PETase for short, and used with the wild-type (WT) in the following kinetic case study.

**Table 1:** Denaturation temperatures of *Is*PETase mutants.

Variant	Melting temperature (mean ± st. dev., n = 3)
Wild-type	48.1 ± 1.3 °C
R280A	48.7 ± 1.2 °C
R280A N233C S282C^a^	62.8 ± 0.4 °C
R280A N233C S282C S121E D186H (TS-PETase)^a^	69.4 ± 0.3 °C

^a^Novel variants generated in this study.

### “Bulk” UV spectrophotometry to estimate PET degradation products formation

The release of MHET, TPA, and BHET from PET substrates have been traditionally detected with HPLC using ultra-violet (UV) absorbance due to the presence of the aromatic ring^4,7,11,12,13^. We propose a bulk absorbance method using a spectrophotometer, where all degradation products contribute to absorbance. The only equipment required is a NanoDrop or a multi-well UV spectrophotometer; the results presented in this paper were obtained using the NanoDrop 1000 (ThermoFisher Scientific), and reactions were performed in 1.5 mL microfuge tubes. When using a multi-well spectrophotometer, reactions can be performed in 1 cm-gap UV-transparent cuvettes and absorbance directly measured (the PET pellet will settle at the bottom and not interfere with the UV path). This method provides bulk measurements similar to the NaOH titration method, as both methods cannot distinguish between the various degradation products. The foremost advantage of the spectrophotometric method is the minimal time and volume required to determine relative product concentrations between enzyme reactions (1.5 µL per measurement with a NanoDrop), making it a method amenable to continuous monitoring of enzyme kinetics in the same reaction vessel over time. Similarly, enzymatic digestion of PET with any PETase homolog releases aromatic products, allowing universal application of this method to assay activity of all PETase homologs.

**Figure 2:**
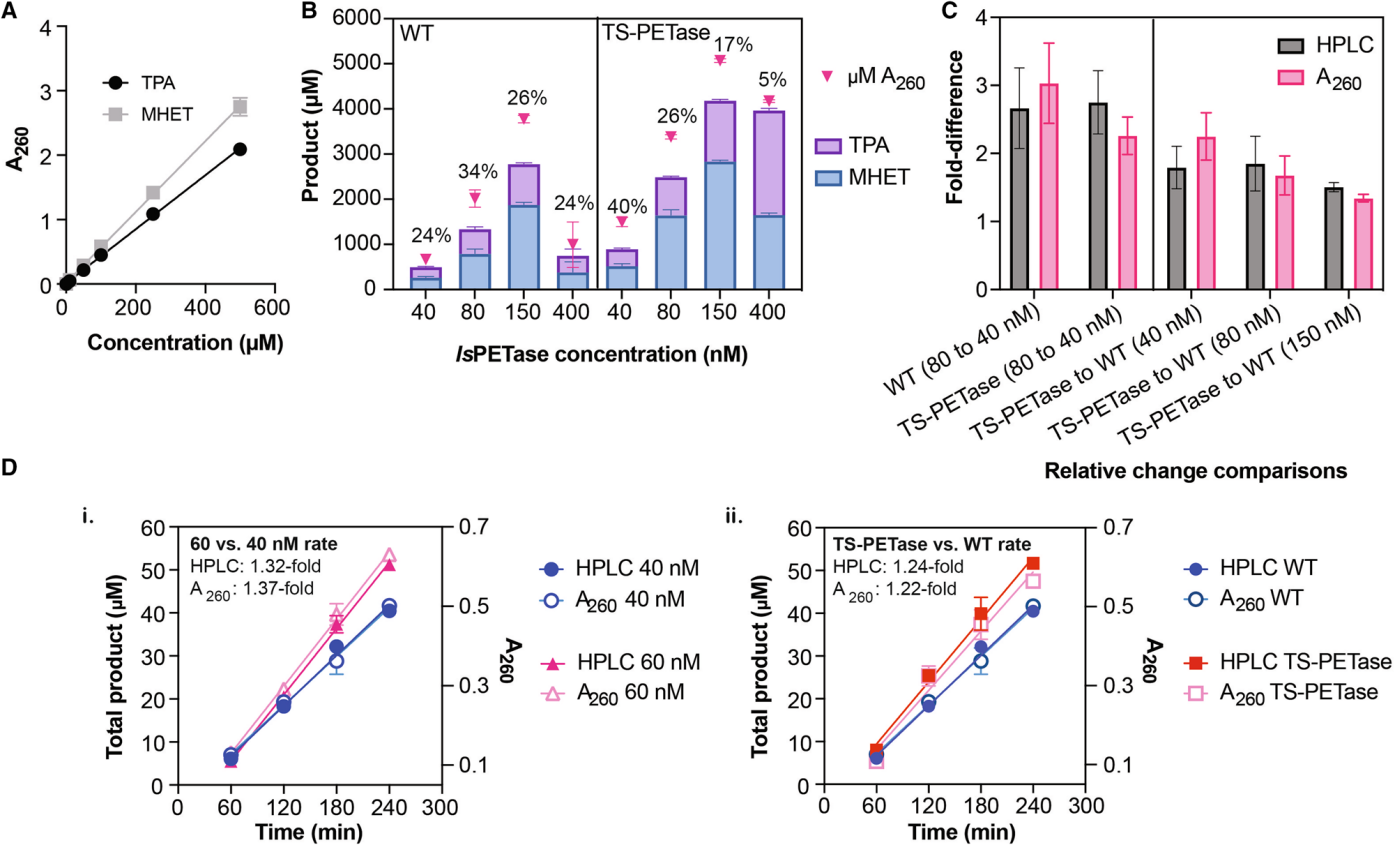
Validation of the absorbance method to measure *Is*PETase activity. (**A**) Absorbance profile of TPA and MHET as measured by NanoDrop 1000 (based on path length of 1 cm). Each concentration was measured in triplicate and error bars show standard error of mean (SEM). (**B**) HPLC-determined product concentrations compared to bulk absorbance estimation using the MHET extinction coefficient for the wild-type (WT) and TS-PETase. Reactions were performed at 30 °C for six days. Bars represent HPLC results, pink triangles are product concentrations estimated from bulk absorbance at A_260_. All points represent mean (*n* ≥ 3) and error bars show SEM. Percentage of additional product estimated by bulk absorbance is indicated above each enzyme concentration (in terms of total estimated product by bulk absorbance). (**C**) Fold-difference in total products for data presented in panel B between concentrations and between *Is*PETase variants as measured by HPLC or bulk A_260_. “Condition 1” to “Condition 2” indicates that fold-difference was calculated as mean of “Condition 1” divided by mean of “Condition 2”. Standard deviation was calculated using ratio distribution. (**D**) Initial rates of *Is*PETase variants incubated with PET film as measured by HPLC and A_260_ (values based on path length of 1 cm) at 30 °C. Triplicate enzyme reactions were performed for each time point and variant; error bars show standard error of the mean. At each time point, the products in the supernatant of the same reaction were measured by both HPLC and bulk absorbance. The interpolated rates from four time points based on HPLC and A_260_ are presented for (i) 40 and 60 nM of wild-type *Is*PETase (WT) and (ii) 40 nM of WT and TS-PETase. Fold-difference between reaction rates determined by HPLC or A_260_ are included for each panel, calculated as “rate of condition 1” divided by “rate of condition 2” for each method. All enzyme reactions were performed at 30 °C. Amounts of TPA, MHET, and BHET can be found in Supplementary Table 1.

The “bulk” absorbance method was first validated on UV spectrophotometers. Absorbance measurements between 240 and 260 nm have been typically used for detection of TPA, MHET, and BHET^4,7,9,11,12,13,22^. The enzyme reaction buffer in this study, however, contains DMSO, which absorbs strongly between 220–250 nm but negligibly at 260 nm (Supplementary Fig. 2). Therefore 260 nm was selected as the detection wavelength for the bulk absorbance method. Linear absorbance profiles at 260 nm were established for TPA and MHET, which are the major products of the reaction (Fig. 2A, Supplementary Fig. 3). Absorbance spectra between 220–350 nm for TPA and MHET are shown in Supplementary Fig. 4. Based on the absorbance profiles, MHET and TPA have distinct extinction coefficients at 260 nm of 5500 M^−1^ cm^−1^ and 4200 M^−1^ cm^−1^, respectively. According to the Beer-Lambert Law, the extinction coefficient of any combination of these products must fall between these two values. Total product estimated from the bulk absorbance method can be under- or overestimated up to 1.3-fold if using the MHET or TPA extinction coefficient, respectively, when the reaction primarily contains one of the two product species. Any combination of TPA and MHET in the reaction, however, should result in a more accurate estimation using either extinction coefficient.

The estimated product concentrations from the spectrophotometry method were validated against HPLC results. All reactions were performed in 1 mL of reaction buffer (50 mM glycine-NaOH pH 9, 50 mM NaCl, and 10% DMSO v/v) with ¼”-diameter amorphous circular PET pellets (total surface area: 0.64 cm^2^). The specific substrate was chosen as it has been used most widely in PET film degradation studies^4,5,16,21,22,31,33^; DMSO was added to solubilize products and for consistency with reaction conditions for BHET hydrolysis assays previously performed in literature^11,12^. Day six product concentration measurements (end-point) were taken using bulk absorbance and HPLC for both *Is*PETase variants at multiple enzyme concentrations. The absorbance method is shown to consistently overestimate the amount of product in the supernatant using the MHET extinction coefficient (Fig. 2B). There likely are unidentified degradation products in the supernatant. A time- and enzyme-dependent appearance of three peaks between 24.0 and 26.2 min are observed on the HPLC for both enzyme variants at 30ºC (Supplementary Fig. 5). These peaks are absent in TPA, MHET, and BHET standards and buffer. The area of the peaks suggest that they contribute non-negligibly to A_260_ and are likely responsible for the concentration overestimation by bulk absorbance, where 15–40% of the estimated products are not accounted for based on the sum of TPA, MHET, and BHET if present (varies between 0–8%) from HPLC (Supplementary Table 1).

Nonetheless, bulk absorbance still captures the fold-differences in total accumulated products between enzyme concentrations and variants, as well as relative rates of product formation, validating the bulk absorbance as a method to observe relative enzyme kinetics (Fig. 2C,D). The presence of BHET and the unidentified products do not interfere with the accuracy of the relative absorbance measurements (Fig. 2D, Supplementary Fig. 6, and Supplementary Table 1). It is important to note that the accuracy of the relative measurements can be affected by product composition (for example, when comparing 100% MHET reaction to 100% TPA reaction). As a guideline, bulk absorbance is reliable to determine kinetic profiles of PET hydrolases, such as reaction trajectories and linear regions of reaction, but it can be problematic to use alone to determine changes in activity. HPLC should be used to confirm that bulk absorbance can reliably capture fold-difference between variants of interest by assaying a time point within the linear region, and especially for maximum rate differences below 1.3-fold.

### *Is*PETase behavior and effects of thermostability on *Is*PETase productivity

**Figure 3:**
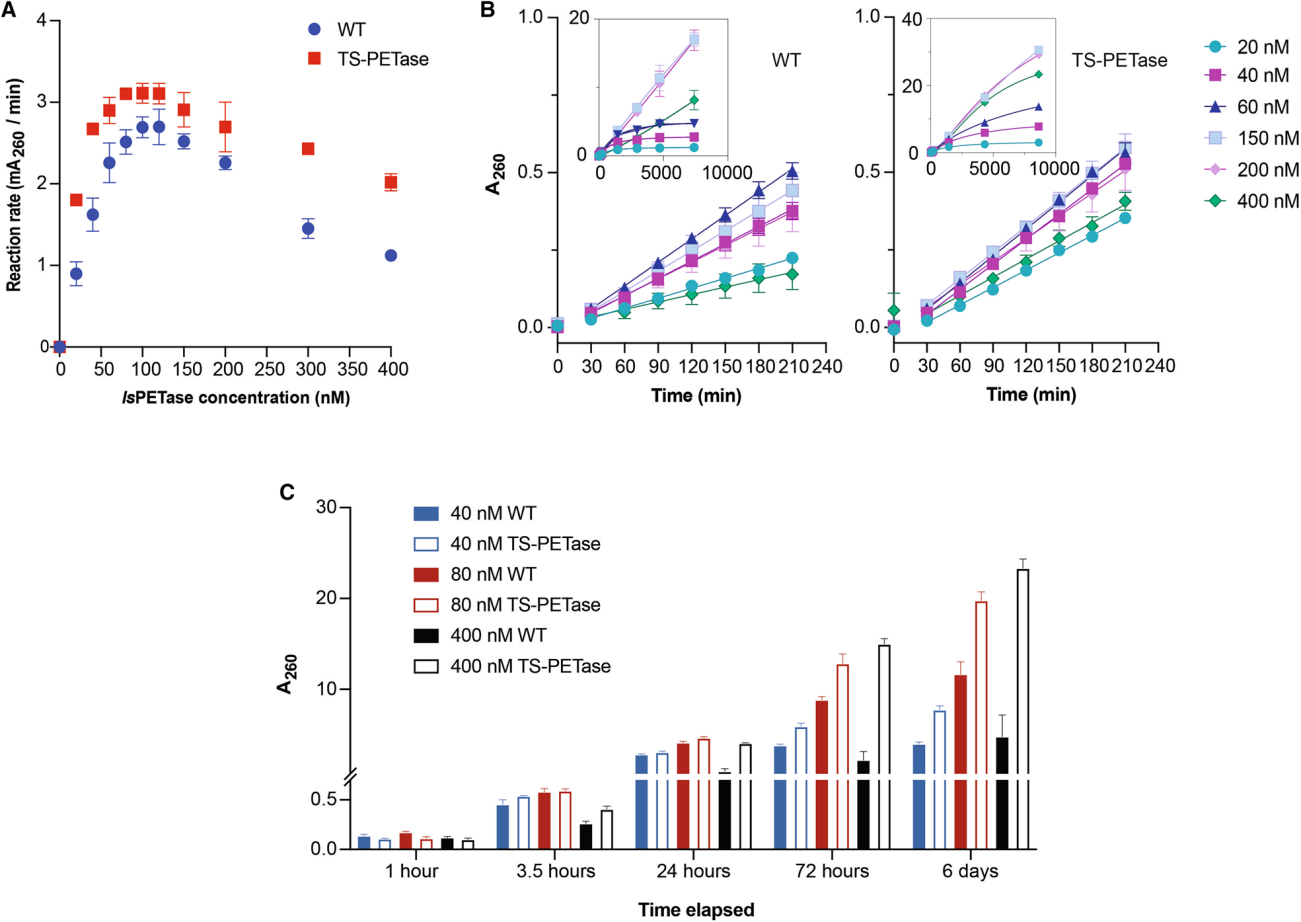
Wild-type vs. TS-PETase enzyme kinetics at 30ºC. (**A**) Plot of initial rates in terms of mA_260_ per minute vs. enzyme concentration for wild-type and TS-PETase (*n* ≥ 4 per concentration). All measurements were performed on a NanoDrop 1000 and values reported with a path length of 1 cm. (**B**) Initial rates determined using bulk absorbance at representative enzyme concentrations (*n* ≥ 4 per concentration); insets display representative product accumulation profiles over extended time (*n* = 3). (**C**) Comparison of product evolution profile by bulk absorbance of wild-type versus TS-PETase over six days (*n* ≥ 3 per time point). Wild-type bars are solid, TS-PETase bars are bordered. Error bars for all panels represent SEM from two or more independent experiments performed on different days.

The effects of enzyme concentration on reaction kinetics was determined using the bulk absorbance method (Supplementary Fig. 7 shows an example of absorbance profiles of a reaction time course). Enzyme-concentration inhibition effect was observed at higher concentrations of *Is*PETase, resulting in a maximum initial catalytic rate between 80 and 120 nM of enzyme for both *Is*PETase variants at 30 °C (Fig. 3A). This is consistent with the original Yoshida et al. report where they showed a decrease in overall product formation at higher enzyme concentrations (maximum production between 50–100 nM enzyme at 18 h)^11^.

The S121E D186H R280A mutations were shown to improve product accumulation up to five-fold compared to the wild-type at 72 h and 30 °C^7^, and attempts to capture this behavior over time was made using the absorbance method. Interestingly, the five-fold improvement was not observed in initial catalytic rates, though the mutational background now contains an additional disulfide bond (Fig. 3A). Both TS-PETase and wild-type display linear initial rates, but the reaction eventually slows down (Fig. 3B). The timing of the slow-down is concentration and variant dependent; the general trend shows a delayed decrease in product formation rate with increasing enzyme concentration (Fig. 3B). TS-PETase is revealed to have a different velocity vs. enzyme concentration profile compared to the wild-type for both initial rates and long-term product accumulation, suggesting altered enzyme behavior during degradation of PET. TS-PETase exhibits 1.6- to 2-fold higher initial rates at low (20 and 40 nM) and high enzyme concentrations (300 and 400 nM), while the maximum initial velocity achieved by TS-PETase was 20% higher than wild-type based on bulk absorbance (Fig. 3A).

Differences are seen in product evolution profiles between the wild-type and TS-PETase over the course of multiple days, which deviate from initial rate comparisons (Fig. 3C and Supplementary Fig. 8 for day 6 HPLC results). Estimated differences in A_260_ reach 6.8-fold after 72 h and 4.9-fold after 6 days at 400 nM (TS-PETase vs. wild-type). The fold-differences in product accumulation between TS-PETase and wild-type are also higher at 72 h compared to 24 h. These results are consistent with the maximum 5.2-fold difference observed at 72 h reported by Son et al*.*^7^. Conversely, the fold-differences in products observed in the present study between TS-PETase and wild-type at 40 nM and 80 nM of enzyme ranged between 0.6- to 1.9-fold throughout the time course. Together, these results suggest that the large fold-differences observed at late time points between TS-PETase and wild-type are likely due to improved stability instead of improved catalytic rates.

Kinetic experiments over multiple enzyme concentrations and time points have also demonstrated another unknown enzyme behavior in digestion of PET films. At 30 °C, neither higher enzyme concentrations nor initial rates correlate with higher product formation over extended time (Fig. 3A,B). The optimal conditions resulting in the most product accumulation at the end of six days is 150–200 nM enzyme for both the wild-type and TS-PETase.

### Kinetics of TS-PETase at elevated temperatures

**Figure 4:**
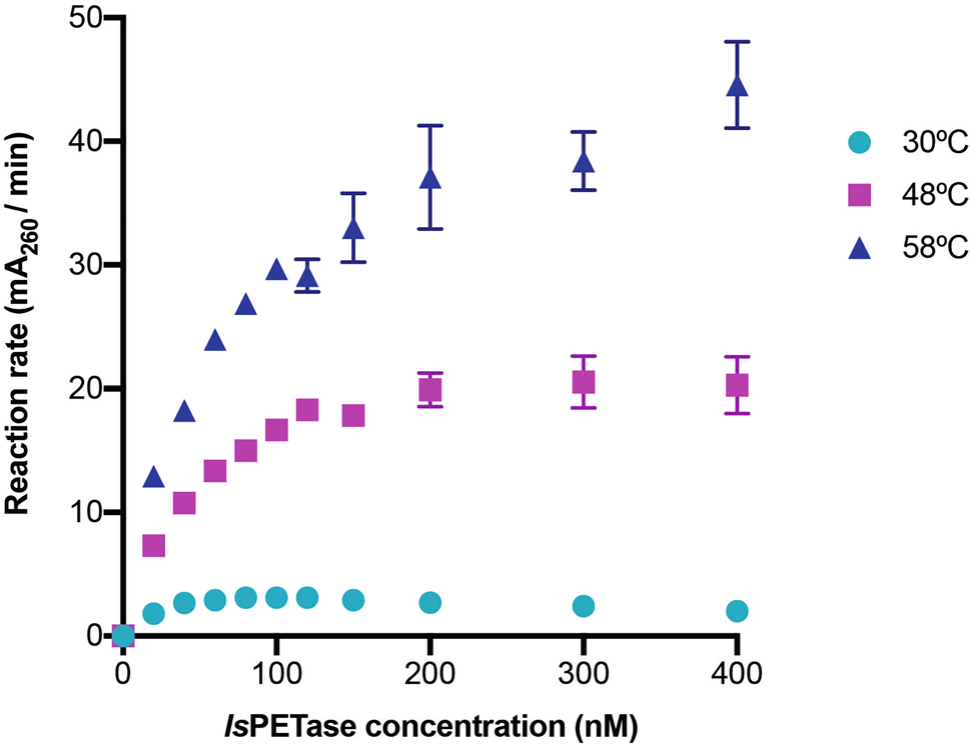
Kinetic profiles of TS-PETase. Initial rates of reaction in terms of mA_260_ per minute at various enzyme concentrations of TS-PETase were determined at 30 °C, 48 °C, and 58 °C (*n* ≥ 4 per time point and concentration). All measurements were performed on a NanoDrop 1000 and values reported with a path length of 1 cm. Error bars represent SEM.

It was important to validate that the bulk absorbance method could detect changes in catalytic rate. Higher temperatures increase activity if an enzyme does not denature during the reaction. Thus, the reaction was performed for TS-PETase at 48 °C and 58 °C to determine initial kinetic rates (Fig. 4, Supplementary Fig. 9). The initial linear velocities increased with each incremental increase in temperature, reaching maximum relative catalytic rate improvements of 6.6- and 14.3-fold at 48 °C and 58ºC, respectively, compared to TS-PETase at 30 °C. This indicates that the absorbance method can reliably capture improvements in catalytic rates, as demonstrated by higher maximum initial rates upon temperature elevation shown in the kinetic curves. In addition, the concentration-dependent inhibition effect of *Is*PETase on catalysis is alleviated at higher temperatures, suggesting a boundary layer phenomenon at the surface of PET that is both temperature and enzyme concentration dependent.

## Discussion

**Figure 5:**
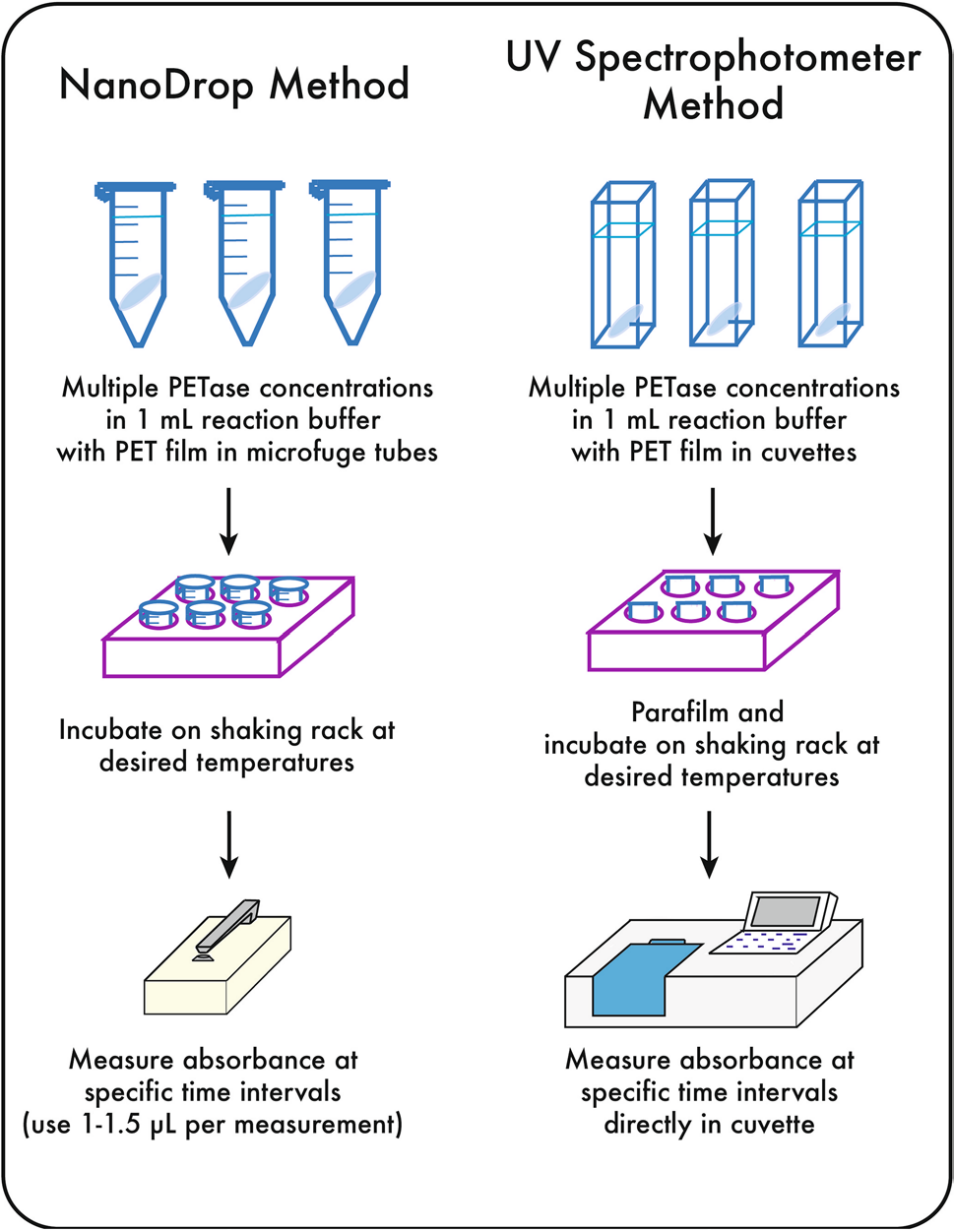
Summary of bulk absorbance method using a NanoDrop (ThermoFisher Scientific) or UV spectrophotometer.

The proposed bulk absorbance assay is a kinetic method to study PET film hydrolysis, where PETase reactions are continuously monitored over the time course of the experiment with UV-absorbance (Fig. 5). This is in contrast to rate determination using different reactions for each time point, which may have more variations from reaction to reaction and potentially resource demanding. Altogether, the bulk absorbance method is quick and easy to perform with minimal resource and equipment requirements, and provides insight into enzyme reaction trajectories and behavior.

The results of the kinetic assays demonstrate how thermostable mutations and *Is*PETase concentration can affect product accumulation over time. It also shows that analyses of product accumulation at 24-h windows are insufficient for full understanding of *Is*PETase hydrolysis of PET. When comparative activity assays between engineered *Is*PETase mutants and the wild-type are performed with sparse measurements, results may be obtained that suggest a substantial improvement in activity of the engineered variant though the catalytic rate was not considerably altered. Such conclusions can frustrate progress in the field due to misattribution of structure–function relationships and can be avoided with careful kinetic studies of the enzyme.

The kinetic results suggest that thermostable variants can have superior performance on PET films, whether at ambient or elevated temperatures, despite having similar intrinsic catalytic potential compared to the wild-type. The wild-type loses activity while TS-PETase continues to hydrolyze the PET film over time, resulting in more total turnovers for TS-PETase at the same concentration of enzyme. An absence of kinetic studies may not identify real changes in catalytic rate and improved product accumulation of TS-PETase may be falsely attributed to improved catalysis rather than stability (the converse can also be true if a variant has higher catalytic potential but is less stable than the wild-type). Thus, the bulk absorbance method provides a simple method for critical analysis of enzyme kinetics, for effects of engineered mutations, and that complements HPLC for exact quantification of product concentrations. Both kinetic and HPLC measurements need to be performed to obtain the overall picture of enzyme behavior.

Furthermore, profiling PETase kinetics on PET films may allow pseudo-quantitative studies of changes in enzyme interactions with the substrate. These changes are likely to be exhibited in shifted catalytic rate vs. enzyme concentration curves, which is observed for WT and TS-PETase, suggesting potentially altered PET binding behaviors. Currently, due to the unusual kinetic profiles for *Is*PETase, no suitable kinetic model can be fitted to the data to extract relative *K*_*D*_ and associated parameters*.* The construction of a suitable model requires more investigation to determine the events occurring during the digestion of PET films by *Is*PETase.

We have presented here a fast, simple, and universal method to study PET degradation kinetics, and demonstrated the importance of kinetic studies to reveal enzyme behaviors that will not be observed using a small number of end-point measurements and enzyme concentrations. The absorbance method is a relative method for kinetic profiling of PETase reactions that provides important insights into enzyme behaviors over time, and HPLC reveals product composition and allows exact quantification. Together, these data will provide critical information to understand PETase behaviors at the solid PET surface.

## Materials and methods

### Structural alignment

MacPyMOL (ver 1.8.6.2) was used to perform the structural alignment with the “align to molecule */CA” function to generate Fig. 1^32^. The wild-type *Is*PETase structure used for the alignment was from Austin et al.^13^ (PDB 6EQE) and the Cut190* structure used was from Oda et al.^5^ (PDB 5ZNO).

### *Is*PETase cloning and mutagenesis

Wild-type *Is*PETase was codon optimized for expression in *Escherichia coli* and purchased from Invitrogen (ThermoFisher Scientific), which was cloned using NEBuilder HiFi DNA Assembly Master Mix (New England BioLabs) into pET-21a without its native secretion signal peptide and with a C-terminal His_6_-tag (sequence found in Supplementary Fig. 10). All mutations were introduced into the wild-type *Is*PETase using targeted mutagenesis primers. All cloning primers can be found in Supplementary Table 2. Briefly, primers were designed to contain the desired mutation and then used to amplify the entire plasmid backbone and insert for 18 cycles using the Phusion High-Fidelity DNA Polymerase (New England BioLabs) with an annealing temperature of 58ºC^34^. The PCR reaction was digested with DpnI for 6 h at 37 °C and then transformed into TOP10F’ *E. coli* cells (New England BioLabs). All successful clones were sequence verified.

### *Is*PETase purification

All variants were purified in a similar manner as Yoshida et al.^11^. Briefly, pET-21a-*Is*PETase was transformed into *E. coli* T7 Express cells. Transformants were picked off the plate and cultured overnight at 37 °C to generate the starter culture, which was diluted 1:100 into Terrific Broth the next day. The cells were cultured for 2–3 h at 37 °C until OD600 ≈ 0.6 and then induced with 1 mM Isopropyl β-d-1-thiogalactopyranoside (IPTG) and cultured overnight at 16 °C. Cells were harvested via centrifugation at 4000 rpm and lysed using sonication in resuspension buffer (50 mM glycine-NaOH pH 9, 50 mM NaCl, 20 mM imidazole). Note that 50 mM NaCl was added because we found it to be the optimal salt concentration for maximum enzyme activity. The cell lysate was centrifuged at 10 000 rpm for 30 min, filtered through a 0.22 μm filter, and then incubated with Ni–NTA (ThermoFisher Scientific) resin overnight at 4ºC. The resin was washed five times with 10 mL of resuspension buffer and then eluted with 50 mM glycine-NaOH pH 9, 50 mM NaCl, and 300 mM imidazole. The elution was first passed through a 50 kDa cutoff filter to remove large aggregates and then concentrated in a 10 kDa cutoff concentrator. Buffer exchange into 50 mM glycine-NaOH pH 9 and 50 mM NaCl buffer was performed simultaneously. Final purified protein was quantified using Bio-rad DC protein assay and stored at 4ºC. All kinetic measurements were performed within 3 days of protein purification.

### Thermostability measurements

All thermostability measurements were performed using differential scanning fluorimetry with Sypro-Orange dye (ThermoFisher Scientific) on the Eppendorf Mastercycler Realplex. 0.5–1 mg per mL protein was mixed with 1:500 Sypro-Orange dye with a final volume of 10 μL.

### Kinetic measurements

All enzyme reactions were performed in duplicate in 1 mL in 1.5 mL microcentrifuge tubes (GeneMate) with the same reaction buffer (50 mM glycine-NaOH pH 9, 50 mM NaCl, 10% DMSO v/v). PET films were purchased from Goodfellow USA (250 μm thick, amorphous, product number ES301445) and cut into ¼” diameter circular pellets using a hole puncher (available surface area: 0.64 cm^2^). The tubes were placed on a shaking rack at 200 rpm and incubated at desired temperatures. A blank control of reaction buffer with PET film was done for instrument blanking and background subtraction. For kinetic reactions performed at 30 °C, 1.5 μL samples were taken every 30 min for 210 min and measured at 260 nm on the NanoDrop 1000 (ThermoFisher Scientific). The values reported on the NanoDrop 1000 are based on a path length of 1 cm. When absorbance approached the detection limit of the instrument, dilutions were made accordingly for measurement. Measurements were taken every 15 min for assays performed at higher temperatures; enzyme concentrations were assayed in small batches such that the temperature of the reaction is maintained during measurement.

### Reversed-phase HPLC

HPLC was performed using an Agilent C18 column (Eclipse XDB-C18, Agilent). Mobile phases and run conditions were as described by Yoshida et al.^11^. The gradient times were as follows: 0 to 15 min (25% methanol, 75% 20 mM phosphate pH 2.5), 15 to 25 min (gradient from 25 to 100% methanol), 25 to 28 min (gradient from 100 to 25% methanol), 28 to 32 min (25% methanol, 75% 20 mM phosphate pH 2.5). Samples were prepared by diluting 1:1, 1:4, or 1:9 with 40 mM phosphate buffer pH 2.5 and heated at 85ºC for 15 min, or heated undiluted depending on the product concentrations. All samples were filtered through a 0.22 µm filter (PES, VWR). 2 or 5 μL of each sample was injected onto the column. Amounts of products were quantified using standard curves shown in Supplementary Fig. 11.

## Supplementary Information

Supplementary Information.

## Data Availability

All datasets generated during and/or analyzed during the current study are available from the corresponding author on reasonable request.
