# Supplementary material for: An absorbance method for analysis of enzymatic degradation kinetics of poly(ethylene terephthalate) films
Source: Sci Rep. 2021 Jan 13;11:928. doi: 10.1038/s41598-020-79031-5 (PMC7806724; doi:10.1038/s41598-020-79031-5)
Supplement: Supplementary file 1 — Supplementary Information. [file 41598_2020_79031_MOESM1_ESM.pdf]

Supplementary Information

# An absorbance method for critical analysis of enzymatic degradation kinetics of poly(ethylene terephthalate) films

En Ze Linda Zhong-Johnson, Christopher A. Voigt, Anthony J. Sinskey*

*Correspondence addressed to: asinskey@mit.edu

[1]Department of Biology, Massachusetts Institute of Technology, Cambridge, MA, USA

[2]Department of Biological Engineering, Massachusetts Institute of Technology, Cambridge, USA

This PDF contains:

Supplementary Figures 1-11

Supplementary Table 1-2

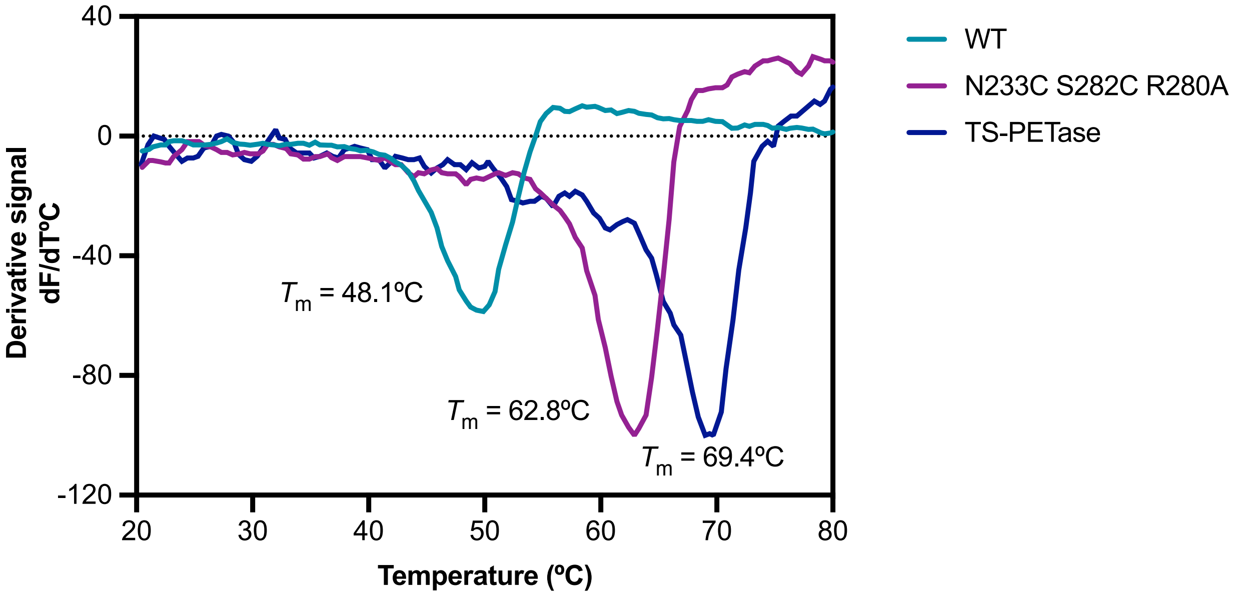


**Supplementary Figure 1. First derivatives of denaturation curves of *Is*PETase variants as measured by differential scanning fluorimetry.** Representative first derivatives of denaturation curves are shown. Average $T_m$ is shown next to the curves for the various variants.

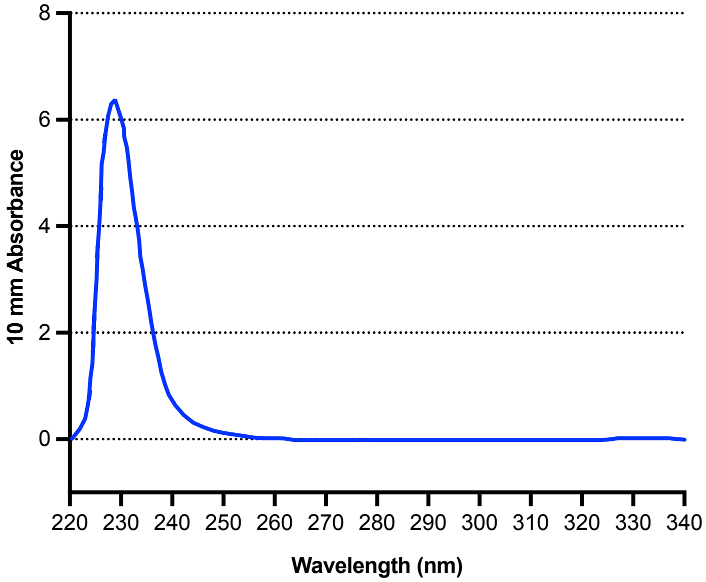


**Supplementary Figure 2. Absorbance profile of DMSO in 50 mM glycine-NaOH pH 9, 50 mM NaCl, 10% DMSO (v/v).** Instrument was blanked with 50 mM glycine-NaOH pH 9, 50 mM NaCl before taking the measurement.

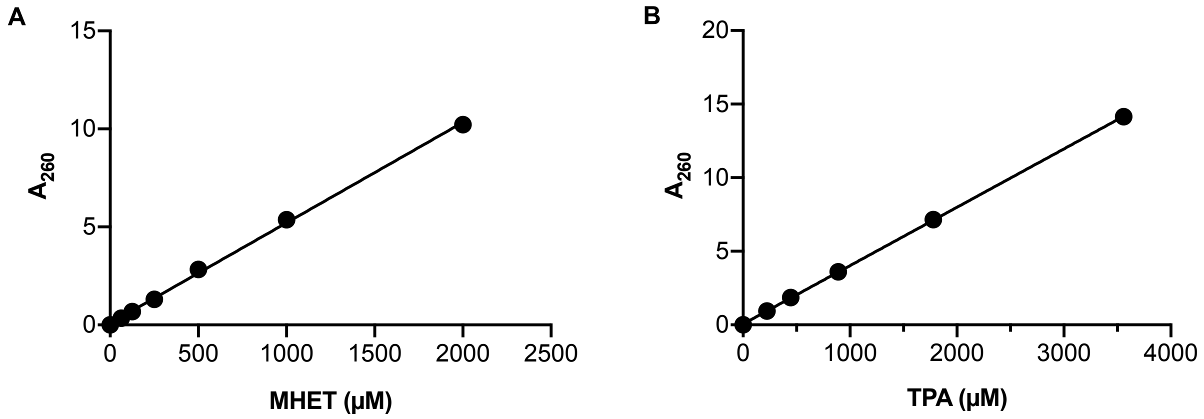


**Supplementary Figure 3. Extended linear response profile of MHET and TPA as measured on the NanoDrop 1000 (ThermoFisher Scientific).** Each point represents mean $A_{260}$ at each concentration (*n* = 3) and error bars represent SEM. The absorbance values are based on a path length of 1 cm.

A

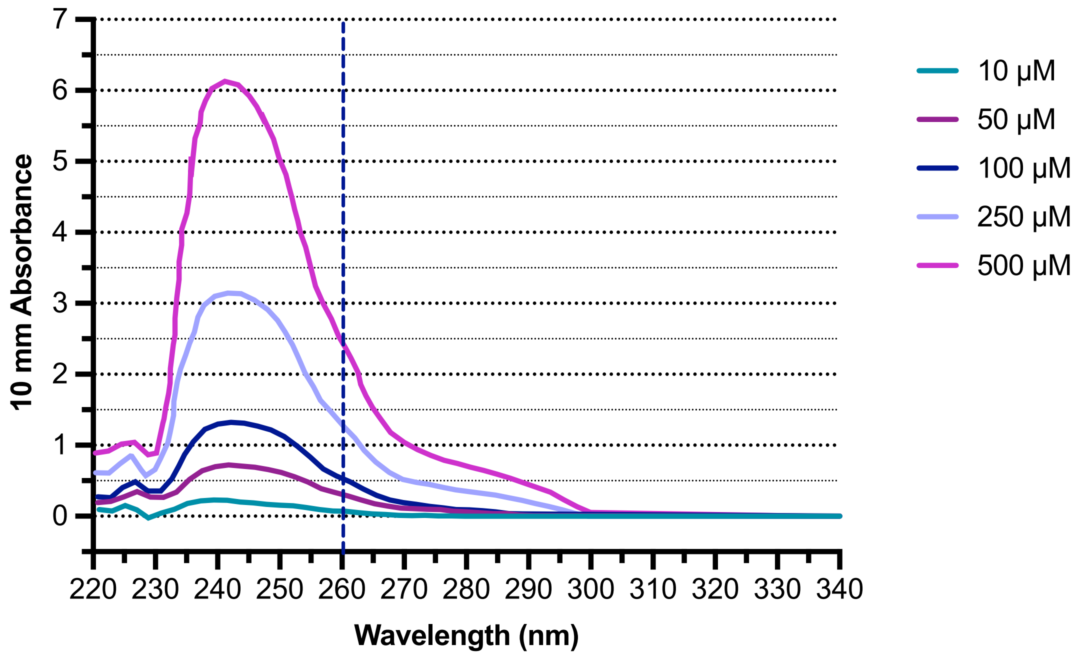


B

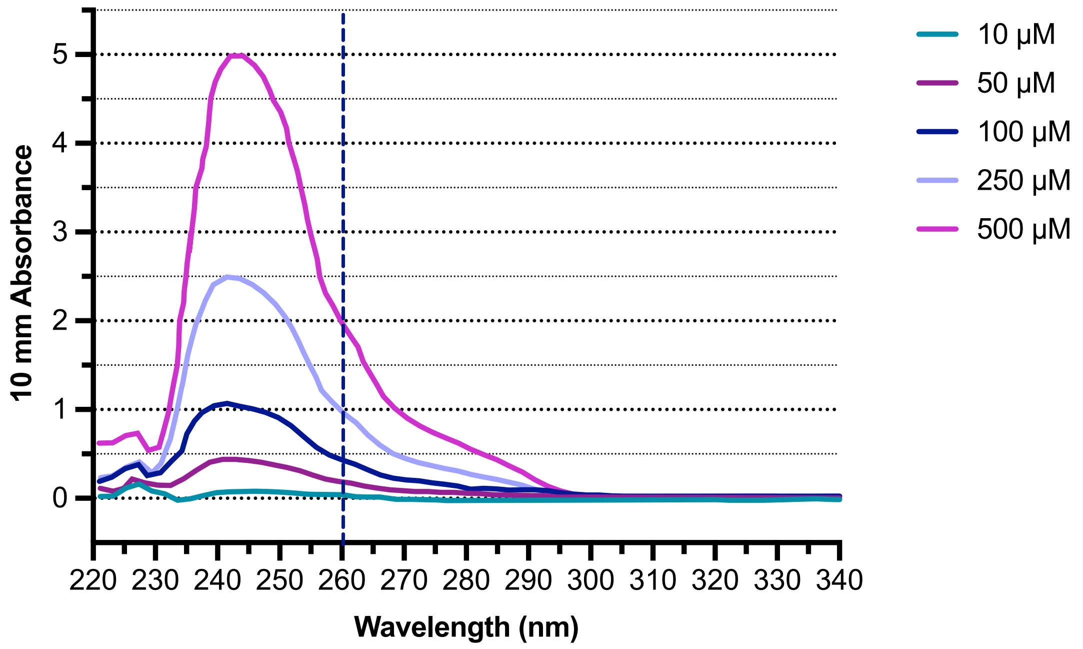


**Supplementary Figure 4. UV-Absorbance profiles for MHET and TPA on NanoDrop 1000.** Absorbance profiles of various concentrations of A) MHET and B) TPA used to generate standard curves. Absorbance is shown from 220 nm to 340 nm.

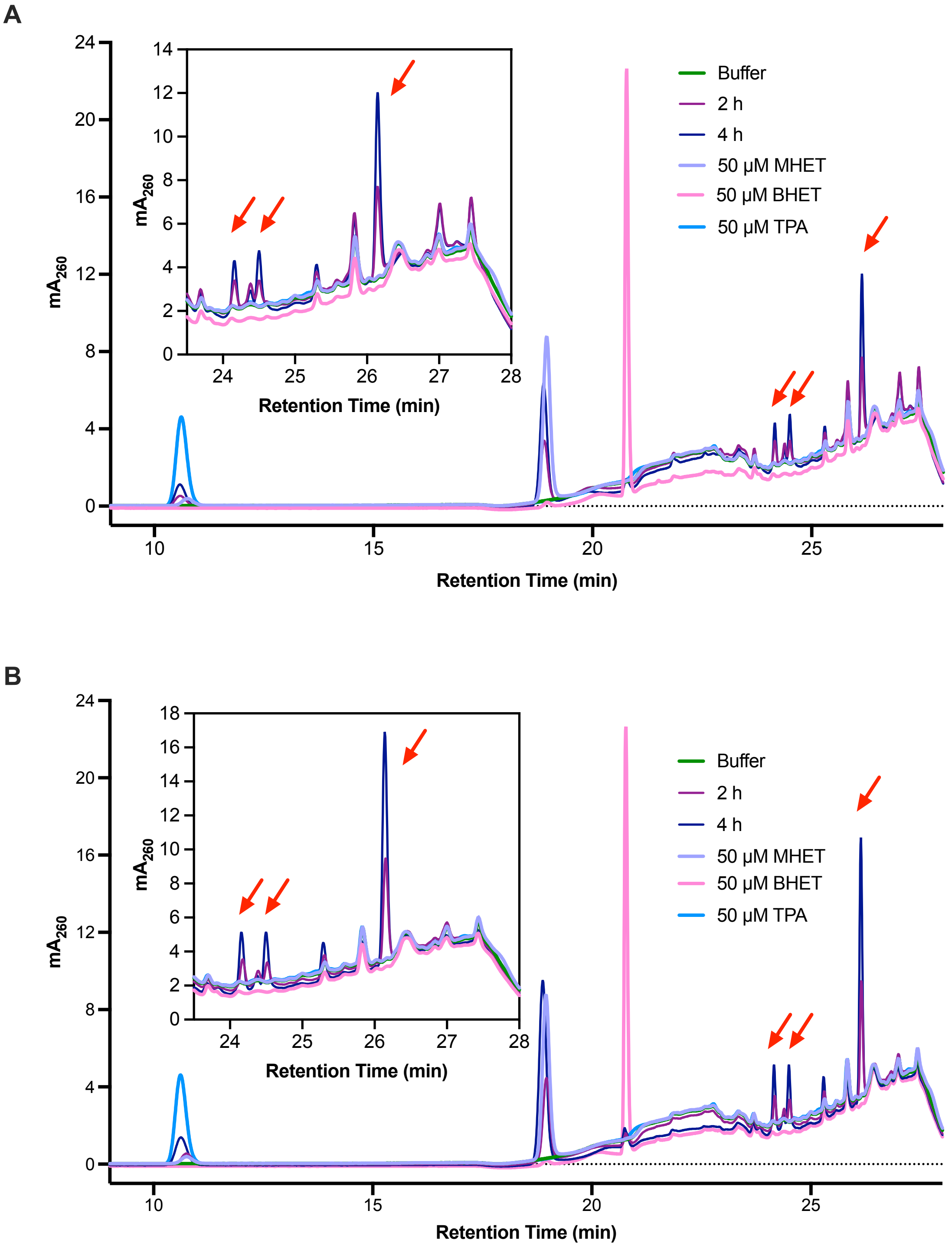


**Supplementary Figure 5. HPLC curves of reaction supernatants from PET films incubated with 80 nM of *Is*PETase variants at 30ºC.** HPLC curves of supernatants of A) wild-type *Is*PETase and B) TS-PETase incubated with PET film at various time points. The red arrows indicate the peaks that appear exclusively in enzyme reactions.

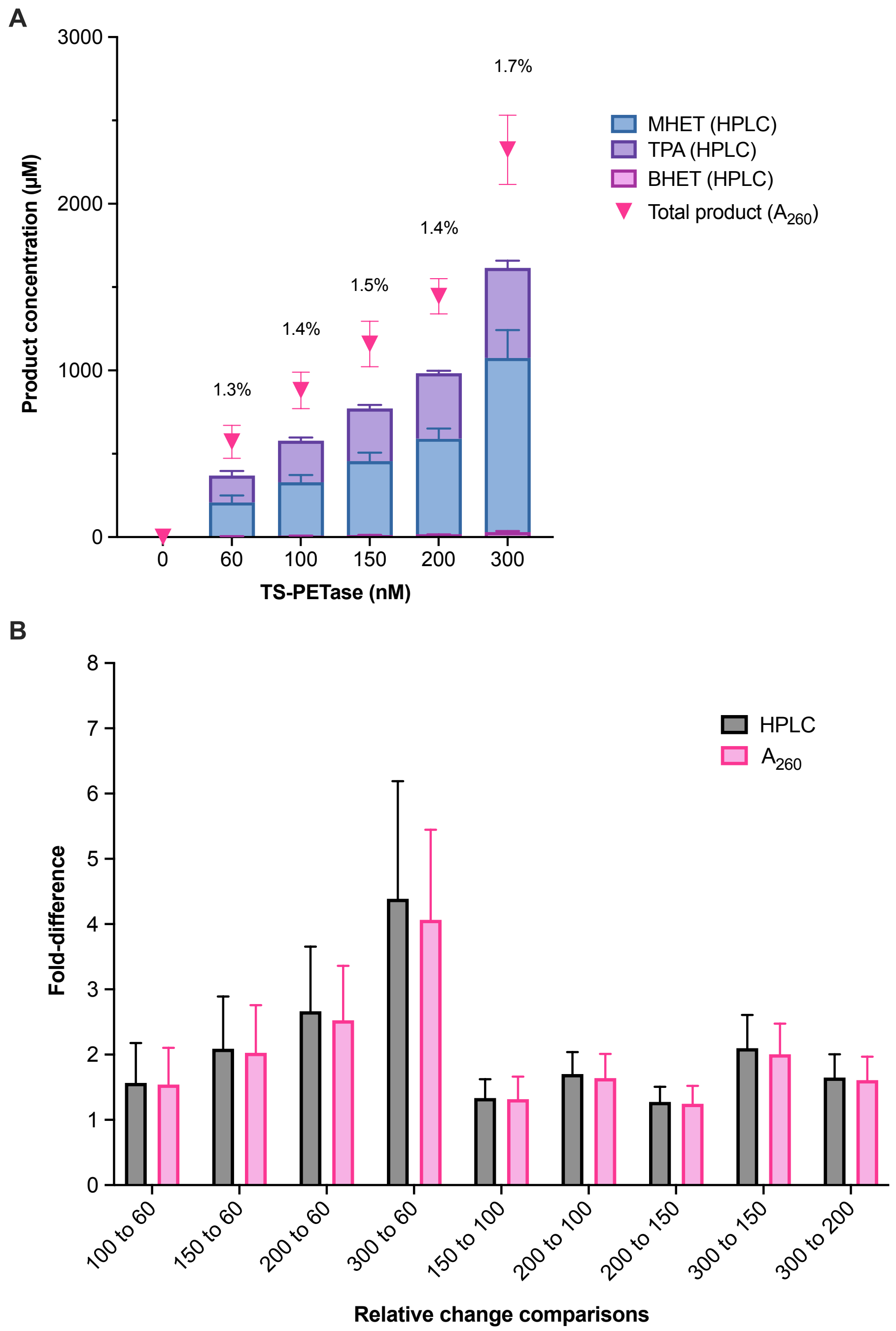


**Supplementary Figure 6. Reaction supernatants of TS-PETase containing BHET.** The reactions with PET film were performed at 58ºC and assayed at 24 hours. A) HPLC quantification and $A_{260}$ estimation of total product concentrations ($n$ = 3) using the MHET extinction coefficient. Error bars show SEM. Average percentage of BHET in the reaction is indicated above each bar. B) Fold-difference in total product accumulated between samples based on HPLC and $A_{260}$ ($n$ = 3 for each concentration). Fold-difference for "Concentration 1" to "Concentration 2" is calculated based on mean of "Concentration 1" divided by "Concentration 2". The error bars show standard deviation calculated using ratio distribution.

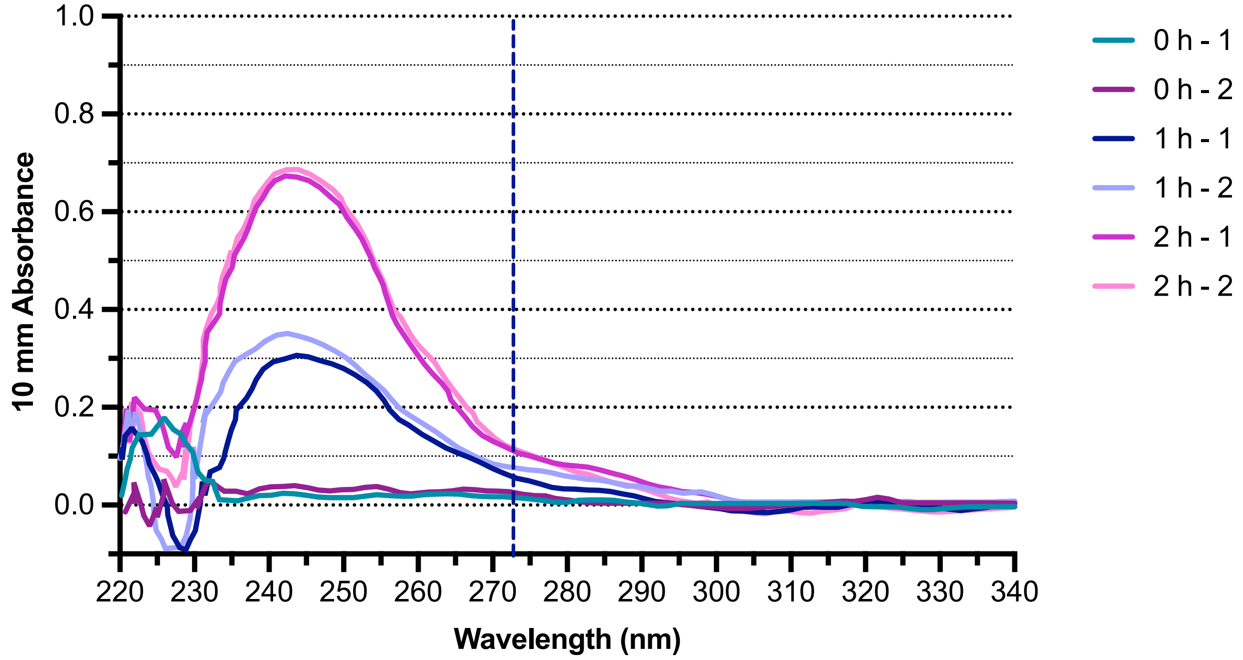


**Supplementary Figure 7. Bulk absorbance profiles of reaction supernatants.** PET films were incubated with 80 nM of *Is*PETase WT in duplicate (indicate as -1 and -2 on figure). Supernatant absorbance profiles were recorded at time 0, 1h, and 2h.

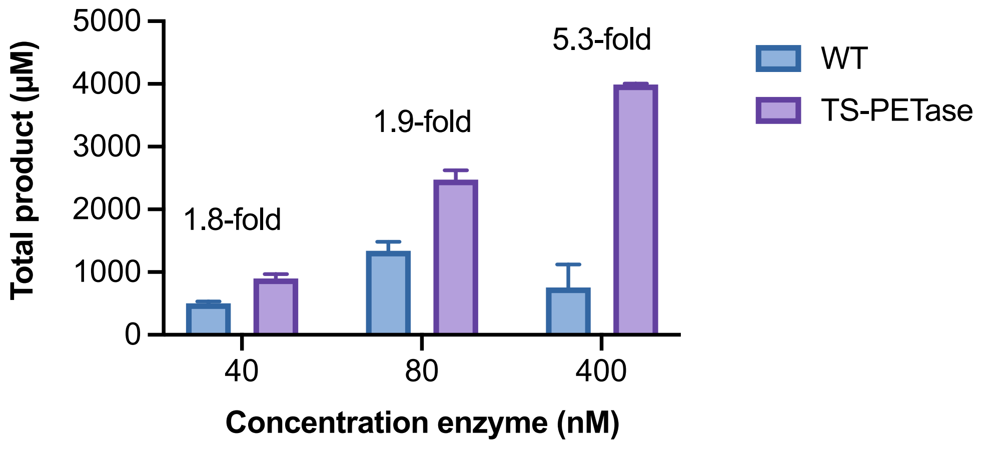


**Supplementary Figure 8. HPLC quantification of products in day 6 enzyme reaction supernatants (30ºC).** Absolute quantification of products in reaction supernatants ($n \geq 3$) for *Is*PETase WT and TS-PETase by HPLC. Fold-difference of total product concentrations between TS-PETase and WT are shown above each set of bars. Error bars represent SEM from two independent experiments.

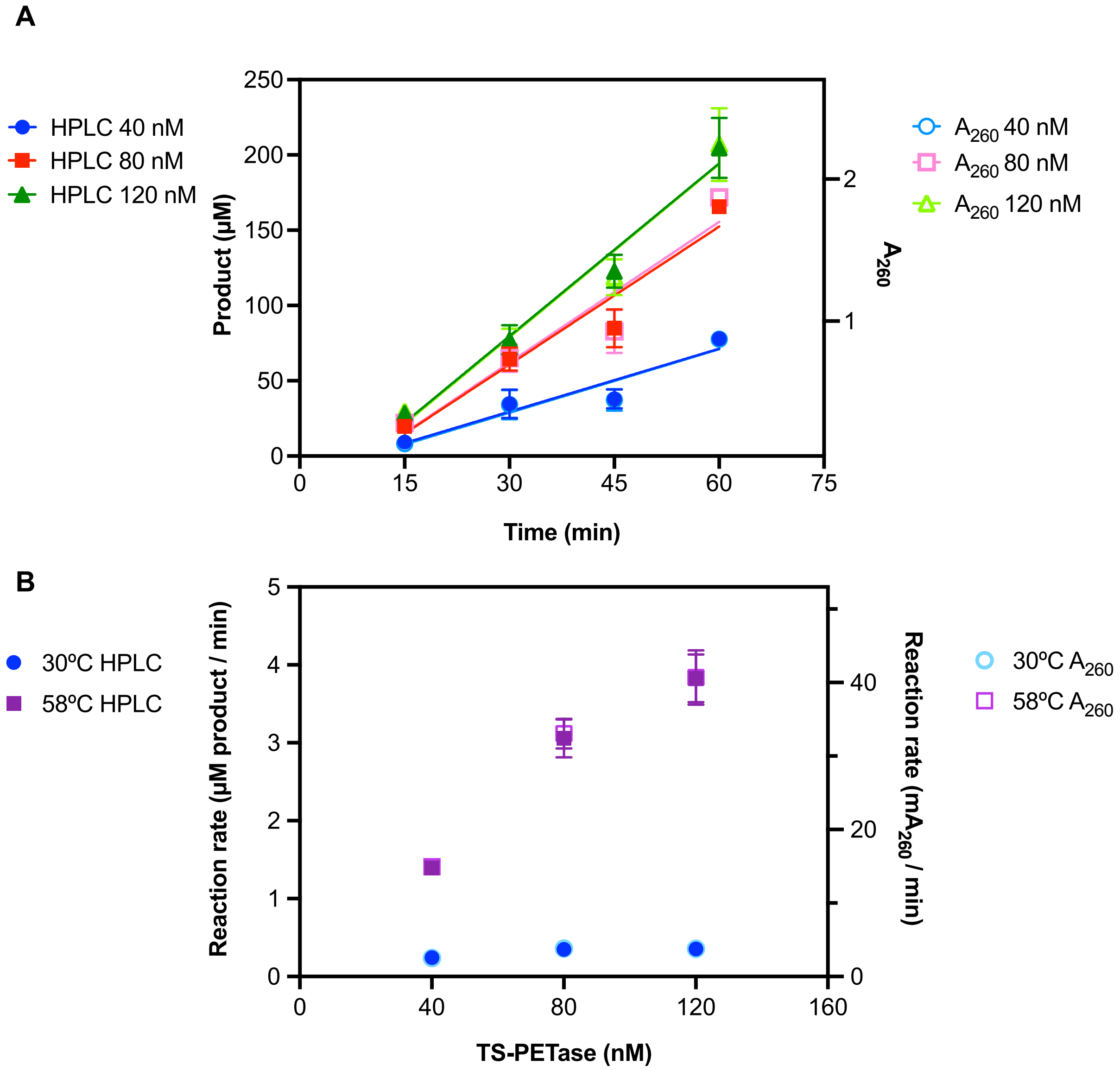


**Supplementary Figure 9. Initial rates of TS-PETase as measured by bulk absorbance and HPLC.** Filled symbols represent HPLC data, open symbols represent bulk absorbance data measured on the NanoDrop 1000 (path length = 1 cm). A) Initial rate curves interpolated from four time points ($n$ = 3 per time point) for TS-PETase incubated with PET films at 58ºC. Each time point is measured in duplicate, and the supernatant of each reaction are measured by both HPLC and bulk absorbance ($A_{260}$). B) Rate vs. enzyme concentration profiles for three TS-PETase concentrations as measured by HPLC and bulk absorbance ($A_{260}$) at 30ºC and 58ºC. Error bars show SEM for both graphs.

```
         10|       20|       30|       40|       50|       60|
                                    MQTN PYARGPNPTA ASLEASAGPF TVRSFTVSRP   60
SGYGAGTVYY PTNAGGTVGA IAIVPGYTAR QSSIKWWGPR LASHGFVVIT IDTNSTLDQP  120
SSRSSQQMAA LRQVASLNGT SSSPIYGKVD TARMGVMGWS MGGGGSLISA ANNPSLKAAA  180
PQAPWDSSTN FSSVTVPTLI FACENDSIAP VNSSALPIYD SMSRNAKQFL EINGGSHSCA  240
NSGNSNQALI GKKGVAWMKR FMDNDTRYST FACENPNSTR VSDFRTANCS LEHHHHHH    298
```

**Supplementary Figure 10. Protein sequence of mature *Is*PETase cloned into p21a.** Sequence is numbered with respect to the start of the signal peptide.

A

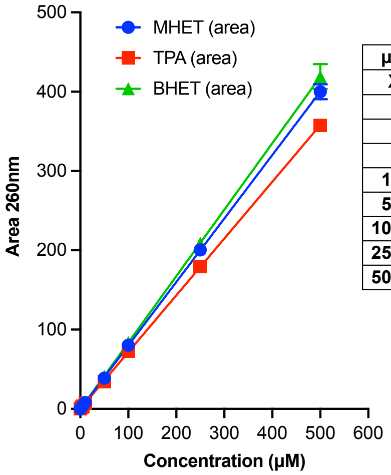


| µM | MHET (area) | | |
|---|---|---|---|
| X | A:Y1 | A:Y2 | A:Y3 |
| **0.0** | 0.0 | 0.0 | 0.0 |
| **3.0** | 2.5 | 2.3 | 2.5 |
| **5.0** | 4.3 | 4.1 | 3.8 |
| **10.0** | 8.1 | 7.6 | 8.6 |
| **50.0** | 38.2 | 39.7 | 38.6 |
| **100.0** | 80.9 | 79.9 | 79.9 |
| **250.0** | 207.6 | 200.3 | 193.8 |
| **500.0** | 417.7 | 396.6 | 385.3 |

| µM | TPA (area) | | |
|---|---|---|---|
| X | B:Y1 | B:Y2 | B:Y3 |
| **0.0** | 0.0 | 0.0 | 0.0 |
| **3.0** | 2.4 | 2.3 | 2.2 |
| **5.0** | 3.1 | 3.8 | 3.8 |
| **10.0** | 7.4 | 6.3 | 7.6 |
| **50.0** | 36.6 | 30.6 | 35.6 |
| **100.0** | 72.9 | 74.6 | 71.9 |
| **250.0** | 181.2 | 178.1 | 179.3 |
| **500.0** | 361.9 | 351.3 | 359.4 |

| µM | BHET (area) | | |
|---|---|---|---|
| X | C:Y1 | C:Y2 | C:Y3 |
| **0.0** | 0.0 | 0.0 | 0.0 |
| **1.0** | 2.1 | 2.0 | 1.9 |
| **2.5** | 5.2 | 5.4 | 4.9 |
| **5.0** | 10.9 | 10.1 | 9.8 |
| **10.0** | 22.4 | 20.5 | 22.3 |
| **50.0** | 113.2 | 103.3 | 106.3 |
| **100.0** | 223.8 | 210.5 | 208.0 |
| **250.0** | 557.0 | 538.2 | 522.5 |
| **500.0** | 1103.6 | 1058.9 | 1067.3 |

B

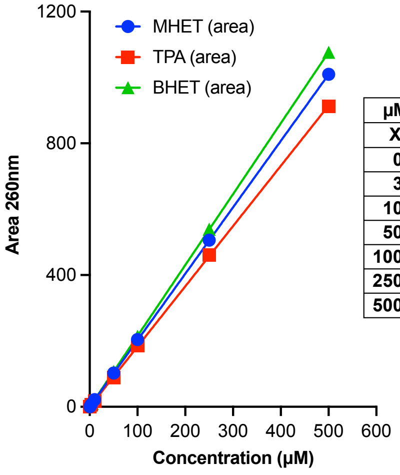


| µM | MHET (area) | | |
|---|---|---|---|
| X | A:Y1 | A:Y2 | A:Y3 |
| **0.0** | 0.0 | 0.0 | 0.0 |
| **3.0** | 6.3 | 7.2 | 6.8 |
| **10.0** | 20.9 | 21.6 | 22.3 |
| **50.0** | 101.4 | 102.8 | 103.8 |
| **100.0** | 204.3 | 202.2 | 205.9 |
| **250.0** | 502.3 | 504.6 | 511.4 |
| **500.0** | 1013.2 | 999.3 | 1015.5 |

| µM | TPA (area) | | |
|---|---|---|---|
| X | B:Y1 | B:Y2 | B:Y3 |
| **0.0** | 0.0 | 0.0 | 0.0 |
| **3.0** | 5.8 | 5.7 | 5.7 |
| **10.0** | 15.0 | 18.7 | 18.6 |
| **50.0** | 80.3 | 92.9 | 92.2 |
| **100.0** | 187.3 | 185.0 | 184.2 |
| **250.0** | 461.2 | 461.5 | 460.5 |
| **500.0** | 916.7 | 914.8 | 907.2 |

| µM | BHET (area) | | |
|---|---|---|---|
| X | C:Y1 | C:Y2 | C:Y3 |
| **0.0** | 0.0 | 0.0 | 0.0 |
| **1.0** | 2.1 | 2.0 | 1.9 |
| **2.5** | 5.2 | 5.4 | 4.9 |
| **5.0** | 10.9 | 10.1 | 9.8 |
| **10.0** | 22.4 | 20.5 | 22.3 |
| **50.0** | 113.2 | 103.3 | 106.3 |
| **100.0** | 223.8 | 210.5 | 208.0 |
| **250.0** | 557.0 | 538.2 | 522.5 |
| **500.0** | 1103.6 | 1058.9 | 1067.3 |

**Supplementary Figure 11. HPLC standard curves for TPA, MHET, BHET (*n* = 3 for each concentration, error bars represent SEM).** A) 2 µL injection standard curves. B) 5 µL injection standard curves. Tables show triplicate values for area under the $A_{260}$ HPLC curves for all three standards.

**Supplementary Table 1. HPLC quantification of TPA, MHET, and BHET in reaction supernatants in Figure 2D and Supplementary Figure 9**

| *Variant and concentration* | Temperature (ºC) | Time (minutes) | Replicate # | MHET (μM) | TPA (μM) | BHET (μM) |
|---|---|---|---|---|---|---|
| *Wild-type 40 nM* | 30ºC | 60 | 1 | 5.9 | 0.0 | 0.0 |
| | | | 2 | 5.7 | 0.0 | 0.0 |
| | | | 3 | 14.7 | 3.9 | 1.0 |
| | | 120 | 1 | 14.4 | 4.0 | 1.0 |
| | | | 2 | 12.4 | 3.4 | 0.0 |
| | | | 3 | 24.4 | 6.6 | 1.8 |
| | | 180 | 1 | 24.1 | 6.7 | 1.6 |
| | | | 2 | 23.2 | 6.6 | 1.7 |
| | | | 3 | 30.5 | 9.0 | 1.9 |
| | | 240 | 1 | 27.7 | 8.6 | 1.8 |
| | | | 2 | 30.7 | 9.5 | 1.8 |
| | | | 3 | 5.9 | 0.0 | 0.0 |
| *Wild-type 60 nM* | 30ºC | 60 | 1 | 6.3 | 0.0 | 0.0 |
| | | | 2 | 5.7 | 0.0 | 0.0 |
| | | | 3 | 4.5 | 0.0 | 0.0 |
| | | 120 | 1 | 14.4 | 4.1 | 0.0 |
| | | | 2 | 15.5 | 4.5 | 0.0 |
| | | | 3 | 17.1 | 4.9 | 1.0 |
| | | 180 | 1 | 25.9 | 7.5 | 1.4 |
| | | | 2 | 30.8 | 8.8 | 1.8 |
| | | | 3 | 26.6 | 7.8 | 1.5 |
| | | 240 | 1 | 36.2 | 11.1 | 1.9 |
| | | | 2 | 37.7 | 11.6 | 2.1 |
| | | | 3 | 39.2 | 11.9 | 2.0 |
| *TS-PETase 40 nM* | 30ºC | 60 | 1 | 7.6 | 0.0 | 0.0 |
| | | | 2 | 10.0 | 0.0 | 0.0 |
| | | | 3 | 6.5 | 0.0 | 0.0 |
| | | 120 | 1 | 19.5 | 4.2 | 0.0 |
| | | | 2 | 22.6 | 4.9 | 0.0 |
| | | | 3 | 18.2 | 5.4 | 1.4 |
| | | 180 | 1 | 36.1 | 8.0 | 2.5 |
| | | | 2 | 32.2 | 7.5 | 0.0 |
| | | | 3 | 25.4 | 6.0 | 1.9 |
| | | 240 | 1 | 41.2 | 9.8 | 2.5 |
| | | | 2 | 37.6 | 9.7 | 2.4 |
| | | | 3 | 39.1 | 9.8 | 2.8 |
| *TS-PETase 80 nM* | 30ºC | 60 | 1 | 12.7 | 0.0 | 0.0 |
| | | | 2 | 13.0 | 0.0 | 0.0 |
| | | | 3 | 6.6 | 0.0 | 0.0 |

| | | | | | | |
|---|---|---|---|---|---|---|
| | | 120 | 1 | 21.9 | 5.4 | 0.0 |
| | | | 2 | 28.7 | 5.7 | 0.0 |
| | | | 3 | 23.6 | 5.7 | 1.6 |
| | | 180 | 1 | 47.7 | 10.9 | 2.7 |
| | | | 2 | 44.9 | 9.5 | 2.5 |
| | | | 3 | 33.4 | 8.3 | 2.1 |
| | | 240 | 1 | 57.2 | 14.4 | 3.3 |
| | | | 2 | 53.7 | 13.6 | 3.2 |
| | | | 3 | 55.1 | 14.0 | 3.3 |
| *TS-PETase 120 nM* | 30ºC | 60 | 1 | 11.0 | 0.0 | 0.0 |
| | | | 2 | 10.5 | 0.0 | 0.0 |
| | | | 3 | 10.5 | 6.3 | 0.0 |
| | | 120 | 1 | 25.7 | 6.6 | 0.0 |
| | | | 2 | 28.0 | 6.3 | 0.0 |
| | | | 3 | 26.4 | 10.4 | 1.8 |
| | | 180 | 1 | 39.0 | 9.4 | 0.0 |
| | | | 2 | 43.9 | 10.3 | 0.0 |
| | | | 3 | 40.7 | 16.8 | 2.2 |
| | | 240 | 1 | 54.0 | 14.2 | 3.0 |
| | | | 2 | 58.7 | 15.6 | 2.9 |
| | | | 3 | 62.7 | 16.8 | 2.9 |
| *TS-PETase 40 nM* | 58ºC | 15 | 1 | 10.9 | 0.0 | 0.0 |
| | | | 2 | 10.1 | 0.0 | 0.0 |
| | | | 3 | 7.0 | 0.0 | 0.0 |
| | | 30 | 1 | 35.5 | 4.6 | 3.3 |
| | | | 2 | 36.6 | 5.0 | 3.1 |
| | | | 3 | 14.5 | 0.0 | 1.3 |
| | | 45 | 1 | 32.5 | 5.2 | 2.6 |
| | | | 2 | 38.6 | 5.9 | 3.2 |
| | | | 3 | 21.1 | 3.2 | 1.7 |
| | | 60 | 1 | 61.1 | 8.7 | 5.7 |
| | | | 2 | 62.5 | 8.8 | 5.6 |
| | | | 3 | 65.9 | 10.2 | 5.2 |
| *TS-PETase 80 nM* | 58ºC | 15 | 1 | 22.4 | 0.0 | 0.0 |
| | | | 2 | 18.5 | 0.0 | 0.0 |
| | | | 3 | 16.8 | 0.0 | 1.3 |
| | | 30 | 1 | 58.1 | 7.3 | 5.3 |
| | | | 2 | 60.4 | 8.0 | 5.1 |
| | | | 3 | 40.1 | 5.7 | 3.4 |
| | | 45 | 1 | 55.9 | 8.8 | 4.2 |
| | | | 2 | 61.2 | 10.0 | 4.7 |
| | | | 3 | 89.1 | 14.0 | 6.7 |
| | | 60 | 1 | 128.7 | 18.7 | 10.7 |
| | | | 2 | 134.2 | 19.7 | 10.6 |
| | | | 3 | 141.7 | 20.7 | 11.7 |

| | | | | | | |
|---|---|---|---|---|---|---|
| *TS-PETase 120 nM* | 58ºC | 15 | 1 | 28.2 | 3.5 | 2.4 |
| | | | 2 | 25.4 | 3.4 | 0.0 |
| | | | 3 | 18.5 | 2.9 | 1.4 |
| | | 30 | 1 | 63.7 | 10.1 | 6.8 |
| | | | 2 | 74.1 | 11.0 | 6.8 |
| | | | 3 | 47.9 | 7.3 | 3.9 |
| | | 45 | 1 | 82.9 | 13.6 | 5.8 |
| | | | 2 | 112.8 | 19.0 | 8.1 |
| | | | 3 | 101.4 | 17.3 | 7.1 |
| | | 60 | 1 | 179.8 | 25.0 | 13.1 |
| | | | 2 | 190.3 | 26.4 | 14.0 |
| | | | 3 | 132.7 | 23.9 | 8.9 |

**Supplementary Table 2. Cloning primer sequences**

| Primer Name | Primer Sequence |
|---|---|
| *Is*PETase F | TTTAAGAAGGAGATATACATATGCAGACCAATCCGTATGCA |
| *Is*PETase R | TGGTGGTGGTGGTGCTCGAGGCTACAATTTGCGGTACGAAAA |
| p21a F | CTCGAGCACCACCAC |
| p21a R | ATGTATATCTCCTTCTTAAAGTTAAACA |
| R280A F | CCGAATAGCACCGCTGTTAGCGATTTTCGTACCGCAAATT |
| R280A R | CGCTAACAGCGGTGCTATTCGGATTTTCGCAGGCAAAG |
| D186H F | CTCCGTGGCATAGCAGCACCAATTTTAGCAGCGTTACCGTTCCGAC |
| D186H R | GTGCTGCTATGCCACGGAGCCTGCGGTGCG |
| N233C F | GTTTCTGGAAATTTGTGGTGGCAGCCATAGCTGTGCAAATAGC |
| N233C R | ATGGCTGCCACCACAAATTTCCAGAAACTGTTTTGCATTACGG |
| R280A S282C F | AAAATCCGAATAGCACCGCTGTTTGTGATTTTCGTACCGCAAATTG TAGCCTCGAG |
| R280A S282C R | CGGTACGAAAATCACAAACAGCGGTGCTATTCGGATTTTCGCAGGC AAAGGTGCT |
| S121E F | CACCCTGGATCAGCCGGAAAGCCGTAGCAGTCAGCAGATGGCAGC ACTGCGTCAGGTTGC |
| S121E R | CATCTGCTGACTGCTACGGCTTTCCGGCTGATCCAGGGTGCTGTTG GTATCAATGGTAAT |
